# Supplementary material for: A porous metal-organic cage liquid for sustainable CO2 conversion reactions
Source: Nat Commun. 2023 Jun 7;14:3317. doi: 10.1038/s41467-023-39089-x (PMC10247695; doi:10.1038/s41467-023-39089-x)
Supplement: Supplementary file 1 — Supplementary Information [file 41467_2023_39089_MOESM1_ESM.pdf]

Supplementary Information

A Porous Metal-Organic Cage Liquid for Sustainable CO_2 Conversion Reactions

Chang He[1,2,3], Yu-Huang Zou[1], Duan-Hui Si[1], Zi-Ao Chen[1,3], Tian-Fu Liu[1,3], Rong Cao[1,3,4]* and Yuan-Biao Huang[1,3]*

[1]State Key Laboratory of Structural Chemistry, Fujian Institute of Research on the Structure of Matter, Chinese Academy of Sciences, Fuzhou 350002, P. R. China

[2]College of Ecological Environment and Urban Construction, Fujian University of Technology, Fuzhou, Fujian 350118, China

[3]University of Chinese Academy of Sciences, Beijing 100049, P. R. China

[4]Fujian Science & Technology Innovation Laboratory for Optoelectronic Information of China, Fuzhou, Fujian, 350108, P. R. China

*Corresponding authors: rcao@fjirsm.ac.cn;ybhuang@fjirsm.ac.cn;

Table of Contents

Section 1. Materials and characterization

Section 2. Syntheses

Section 3. Catalyses

Section 4. Additional Figures and Tables

Section 5. References

Section 1. Materials and characterization

All solvents and reagents were commercially available and used without further purification. Powder X-ray diffraction patterns (PXRD) were recorded on a Rigaku Dmax 2500 diffractometer equipped with Cu-Kα radiation (λ= 1.54056 Å) over the 2θ range of 5-50 ° with a scan speed of 3 ° min^{-1} at room temperature. UV-vis studies were done on a Varian Cary 5000 UV-vis-NIR spectrophotometer at room temperature. Infrared (IR) spectra were recorded using KBr pellets on a PerkinElmer Spectrum One in the range of 400–4000 cm^{-1}. The ^1H NMR was performed at AVANCE III Bruker Biospin spectrometer, operating at 400 MHz. Mass spectra were collected using a Varian 500-MS Ion Trap Mass Spectrometer. Elemental analyses of C, H, and N were carried out on an Elementar Vario EL III analyzer. Elemental analysis results were based on the average of two or more trials for each sample. Thermogravimetric analysis (TGA) was performed at a scan speed of 5 ^oC/min under a stream of nitrogen on a SDT Q600 thermogravimetric analyser. N_2 sorption isotherms for Zn-Cage was measured by using a Micrometrics ASAP 2460 instrument at 77 K. The gravimetric gas solubility measurements of CO_2 were performed on HTP1-V at 298 K for Im-PL-Cage and PEG-imidazolium chains. Before the measurement, the samples were evacuated and activated at 393 K in vacuum for 12 hours. The morphologies of Im-PL-Cage was studied using a FEIT 20 transmission electron microscope (TEM) working at 200 kV and scanning electron microscope (SEM) working at 10 KV. The differential scanning calorimetry (DSC) traces were recorded by a DTA404PC instrument. The rheological properties of Im-PL-Cage were

measured on TA Discovery Hybrid Rheometer-2 at 333 K. The X-ray photoelectron spectroscopy (XPS) analysis of Zn-Cage and Im-PL-Cage were measured on Thermo Fisher Scientific ESCALAB 250Xi.

Single crystal X-ray diffraction datas for Zn-Cage was collected at 100 K using graphite-monochromated Mo-K_{α} radiation (= 0.71073 Å) on a Bruker CCD APEXII diffractometer. The collected frames were processed with the software SAINT. The data were corrected for absorption using the SADABS program.

The simulation of the structure for Im-PL-Cage was modeled and optimized with Forcite Geometry Optimization based on Materials Studio 8.0 package. The simulated pore size distribution of Im-PL-Cage was calculated by Zeo++. The B3LYP hybrid functional were adopted in all calculations by means of the Gaussian16 program, and the 6-31G(d) set as the basis sets. The molecular volume and pore properties of Im-PL-Cage were analyzed by the Multiwfn package, and visualized by VMD program.

Section 2. Syntheses

2.1 Synthesis of [(5-Meim-1,3-H_2BDC)$^+$(Cl^-)][1,2]:

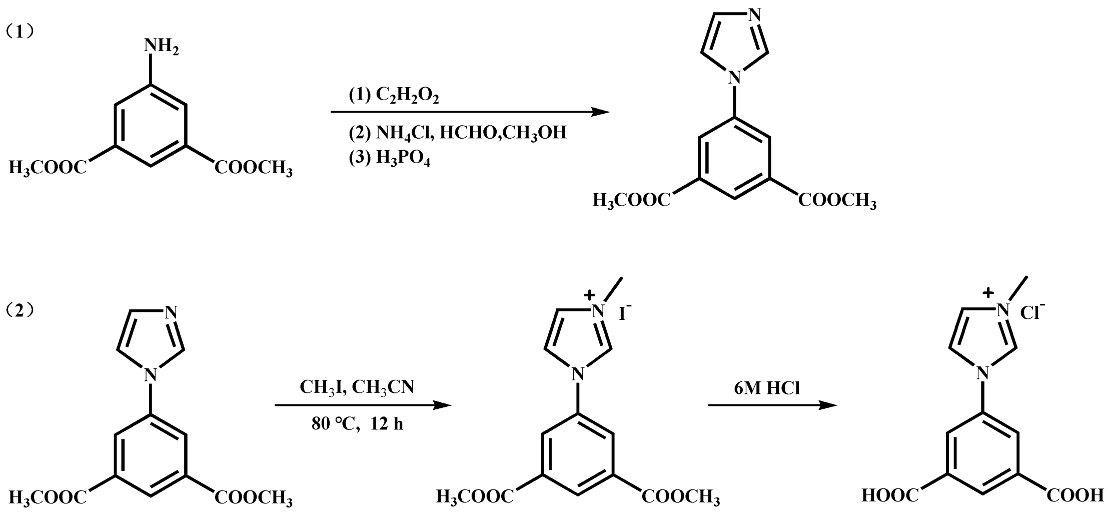

Supplementary Figure 1. The synthesis of [(5-Meim-1,3-H_2BDC)$^+$(Cl^-)].

(1) Synthesis of dimethyl 5-(1H-imidazol-1-yl)-isophthalate

Dimethyl 5-aminoisophthalate (10.49 g, 0.05 mol), 40% oxalaldehyde aqueous solution (5 mL) in 200 mL MeOH were stirred at room temperature for 48 h. NH_4Cl (16.05 g, 0.03 mol) and 37% formaldehyde aqueous solution (16 mL) were stepwise added to the mixture under stirring. The mixture was diluted with 100 mL MeOH, which was refluxed for 1 h. Then, 21 mL H_3PO_4 was added to the mixture dropwise, which was further refluxed for 72 h. The mixture was cooled down to room temperature, and filtered to remove insoluble solid. The filtrate was concentrated under reduced pressure, and poured into 40 mL water. The pH value of the mixture was adjusted to ~9 by 40% KOH aqueous solution, which resulted in large amount of light yellow precipitate. The solid was collected by filtration and washed with water for several times. The product was purified by chromatography on silica gel with hexane/ethyl acetate (2/1) to yield dimethyl 5-(1H-imidazol-1-yl)-isophthalate as a

yellow powder (2.362 g, 9 mmol, 18% yield).^{1}H NMR (400 MHz, DMSO-d_6, ppm): δ = 8.26 (s, 2H), 7.97 (s, 1H), 7.39 (s, 1H), 7.27 (s, 1H), 4.00 (s, 6H). ^{13}C NMR (400 MHz, DMSO-d_6, ppm): δ = 167.4, 137.2, 136.5, 131.3, 130.4, 126.5, 124.8, 123.6, 50.0. HRMS(ESI) m/z: $[M]^+$ calculated for $C_{13}H_{12}N_2O_4$, 260.0795; found, 260.0793. Elemental analysis (calculated, found for $C_{13}H_{12}N_2O_4$): C (60.03, 60.24), H (4.65, 4.57), N (10.76, 10.83).

(2) Synthesis of [(5-Meim-1,3-H_2BDC)$^+$(Cl^-)]

A solution of 5-(1H-imidazol-1-yl)-isophthalate (1.862 g, 7.2 mmol) and CH_3I (13.46 ml, 21.6 mmol) in 50 ml of acetonitrile was heated to reflux and stirred overnight. After cooling the mixture to room temperature, volatiles were evaporated. The obtained residue was rinsed with hexane/ethyl acetate (2/1) to give dimethyl 5-(3-methyl-imidazol-1-yl)-isophthalate as a brown powder (1.58 g, 3.9 mmol, 56% yield). ^{1}H NMR (400 MHz, DMSO-d_6, ppm): δ 9.81 (s, 1H), 8.38 (s, 1H), 8.20 (m, 3H), 7.85 (s, 1H), 3.88(s, 6H), 3.94 (s, 3H). ^{13}C NMR (400 MHz, DMSO-d_6, ppm): δ = 167.9, 138.5, 135.1, 133.9, 130.7, 128.6, 125.9, 124.6, 50.5, 34.2. HRMS(ESI) m/z: $[M]^+$ calculated for $C_{14}H_{15}IN_2O_4$, 402.0077; found, 402.0076. Elemental analysis (calculated, found for $C_{14}H_{15}IN_2O_4$): C (41.81, 41.69), H (3.76, 3.79), N (6.97, 6.93).

Then, in a 50 mL round bottomed flask, dimethyl 5-(3-methyl-imidazol-1-yl)-isophthalate (1.58 g, 3.9 mmol) in 11.4 mL 20% HCl aqueous solution was refluxed for 12 h. The mixture was cooled down to room temperature, and the solvent was removed under reduced pressure to result [(5-Meim-1,3-H_2BDC)$^+$(Cl^-)] in light yellow powder. (0.8 g, 2.84 mmol, 73% yield).

^{1}H NMR (400 MHz, DMSO-d_6, ppm): δ 9.95 (s, 1H), 8.58 (s, 1H), 8.50 (m, 3H), 7.97 (s, 1H), 3.95 (s, 3H). ^{13}C NMR (400 MHz, DMSO-d_6, ppm): δ = 172.5, 140.8, 136.2, 131.7, 131.2, 128.5, 123.7, 35.6. HRMS(ESI) m/z: $[M]^+$ calculated for $C_{12}H_{11}ClN_2O_4$, 282.0413; found, 282.0413. Elemental analysis (calculated, found for $C_{12}H_{11}ClN_2O_4$): C (50.99, 50.65), H (3.92, 3.88), N (9.91, 9.83).

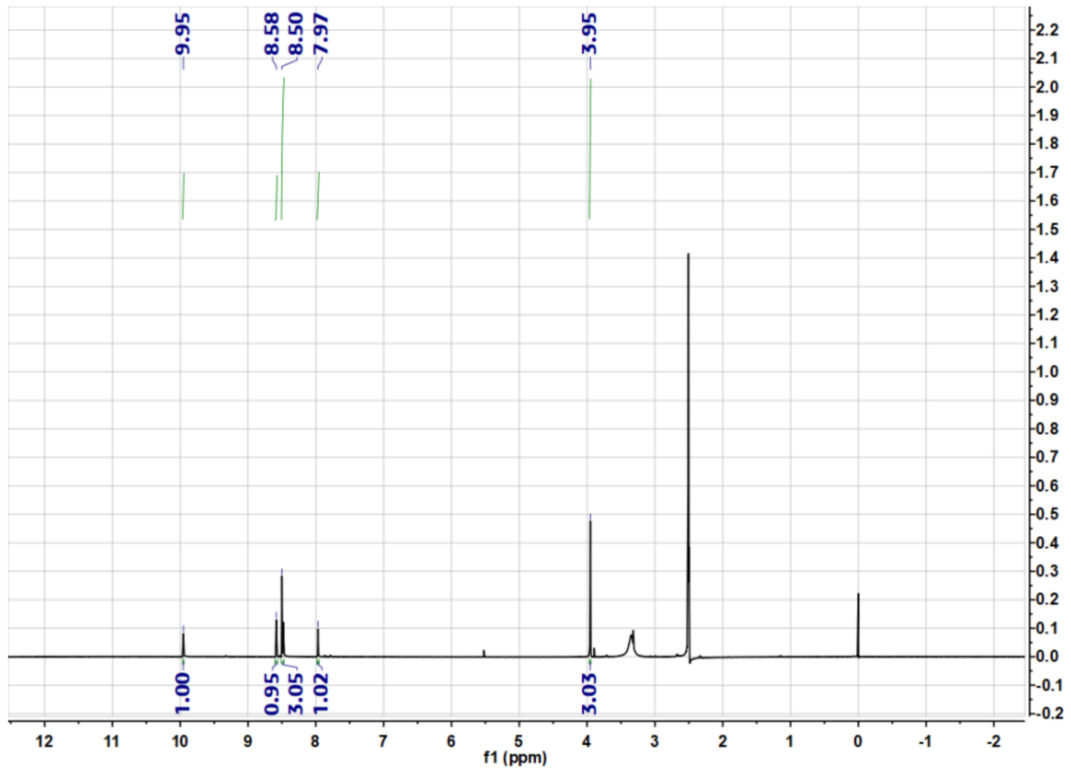

Supplementary Figure 2. ^{1}H NMR spectrum of $[(5\text{-Meim-1,3-}H_2BDC)^{+}(Cl^{-})]$ in DMSO-d_6 (r.t).

2.2 Synthesis of PEG-imidazolium 1,3-benzenedicarboxylic acid (PEG-Im-BDC):

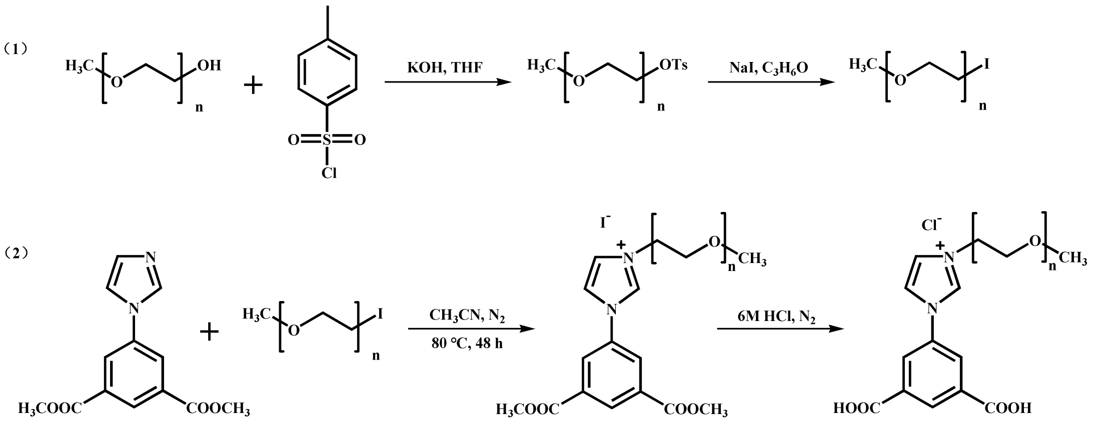

Supplementary Figure 3. The synthesis of PEG-Im-BDC.

(1) Synthesis of iodinated-methoxypolyethylene glycols

A solution of potassium hydroxide (2.24 g, 40 mmol) in water (7.5 mL) was added slowly to a solution of polyethylene glycol (M_w = 4000, 40 g, 10 mmol) and 4-toluenesulfonyl chloride (3.81 g, 20 mmol) in THF (300 ml). The mixture was stirred for 18 h at 273 K. Water (250 mL) was added and the reaction mixture was extracted with DCM (3×100 mL). The organic layer was dried over $MgSO_4$, gravity filtered, and the solvent was removed by rotary evaporation. The desired product 4-methylbenzenesulfonate-methoxypolyethylene glycols was obtained as a white wax/oil (17.38 g, 4.1 mmol, 41% yield). ^{1}H NMR (400 MHz, DMSO-d_6, ppm): δ 7.85 (m, 2H), 7.40 (m, 2H), 3.70 (s, 2H), 3.66 (m, 358H), 3.24 (s, 3H), 2.35 (s, 3H). ^{13}C NMR (400 MHz, DMSO-d_6, ppm): δ = 143.8, 131.7, 130.5, 127.9, 73.2, 70.9, 70.5, 69.7, 62.4, 53.6, 20.8. HRMS(ESI) m/z: $[M]^+$ calculated for $CH_3(OC_2H_4)_nSO_3C_7H_7$ (n = 85-95), 3930.5166, 3974.5692, 4018.6254, 4062.6816, 4106.7378, 4150.7940, 4194.8502, 4238.9064, 4282.9626, 4327.0188, 4371.0750; found, 3930.5168,

3974.5689, 4018.6257, 4062.6813, 4106.7379, 4150.7941, 4194.8501, 4238.9066, 4282.9625, 4327.0187, 4371.0751. Elemental analysis (calculated, found for $CH_3(OC_2H_4)_nSO_3C_7H_7$): C (54.40, 55.75), H (8.98, 8.92).

A solution of 4-methylbenzenesulfonate-methoxypolyethylene glycols (17.38 g, 4.1 mmol) and NaI (12.3 g, 8.2 mmol) in 100 mL of acetone was heated to reflux and stirred stirred under nitrogen overnight. After cooling down to room temperature, water (100 mL) was added and the reaction mixture was extracted with DCM (3 ×50 mL). The organic layer was dried over $MgSO_4$, gravity filtered, and the solvent was removed by rotary evaporation. The product iodinated-methoxypolyethylene glycols was obtained as a yellow wax/oil (15.22 g, 3.7 mmol, 90% yield). ^1H NMR (400 MHz, DMSO-d_6, ppm): δ 3.62 (m, 358H), 3.31 (s, 2H), 3.24 (s, 3H). ^{13}C NMR (400 MHz, DMSO-d_6, ppm): δ = 73.9, 73.2, 70.8, 70.4, 69.6, 53.7, 7.9. HRMS(ESI) m/z: $[M]^+$ calculated for $CH_3(OC_2H_4)_nI$ (n = 85-95), 3886.4158, 3930.4720, 3974.5282, 4018.5844, 4062.6406, 4106.6968, 4150.7530, 4194.8092, 4238.8654, 4282.9216, 4326.9778; found, 3886.4160, 3930.4721, 3974.5283, 4018.5846, 4062.6405, 4106.6970, 4150.7531, 4194.8094, 4238.8653, 4282.9217, 4326.9776. Elemental analysis (calculated, found for $CH_3(OC_2H_4)_nI$): C (52.94, 52.87), H (8.91, 8.84).

(2) Synthesis of PEG-Im-BDC

A solution of dimethyl 5-(1H-imidazol-1-yl)-isophthalate (0.65 g, 2.5 mmol) and iodinated-methoxypolyethylene glycols (4.1 g, 1 mmol) in 50 mL of acetonitrile was heated to reflux and stirred stirred under nitrogen for 48 h. After cooling the mixture to room temperature, volatiles were evaporated. The obtained solid was dissolved in

30 mL 6 M HCl and the mixture was refluxed under nitrogen for 12 hours. Then, the solvent was removed and the mixture was filtered with ethyl acetate (50 mL). The final product PEG-Im-BDC as a dark yellow wax/oil (1.37 g, 0.32 mmol, 13% yield) was obtained by recrystallization from the filtrate at 273K. ^{1}H NMR (400 MHz, DMSO-d_6, ppm): δ 13.87 (s, 2H), 9.99 (s, 1H), 8.54 (m, 4H), 8.03 (s, 1H), 3.51 (m, 360H), 3.24 (s, 3H). ^{13}C NMR (400 MHz, DMSO-d_6, ppm): δ = 172.1, 140.7, 136.5, 131.6, 131.3, 127.8, 127.5, 124.3, 80.2, 73.1, 70.7, 68.6, 53.8. HRMS(ESI) m/z: $[M]^+$ calculated for $C_{11}H_8N_2O_4Cl(OC_2H_4)_nCH_3$ (n = 85-95), 4024.1196, 4068.1758, 4112.2320, 4156.2882, 4200.3445, 4244.4006, 4288.4568, 4332.5130, 4376.5692, 4420.6254, 4464.6816; found, 4024.1195, 4068.1756, 4112.2322, 4156.2885, 4200.3447, 4244.4008, 4288.4566, 4332.5131, 4376.5693, 4420.6255, 4464.6817. Elemental analysis (calculated, found for $C_{11}H_8N_2O_4Cl(OC_2H_4)_nCH_3$): C (54.29, 54.97), H (8.80, 8.53), N (0.66, 0.75).

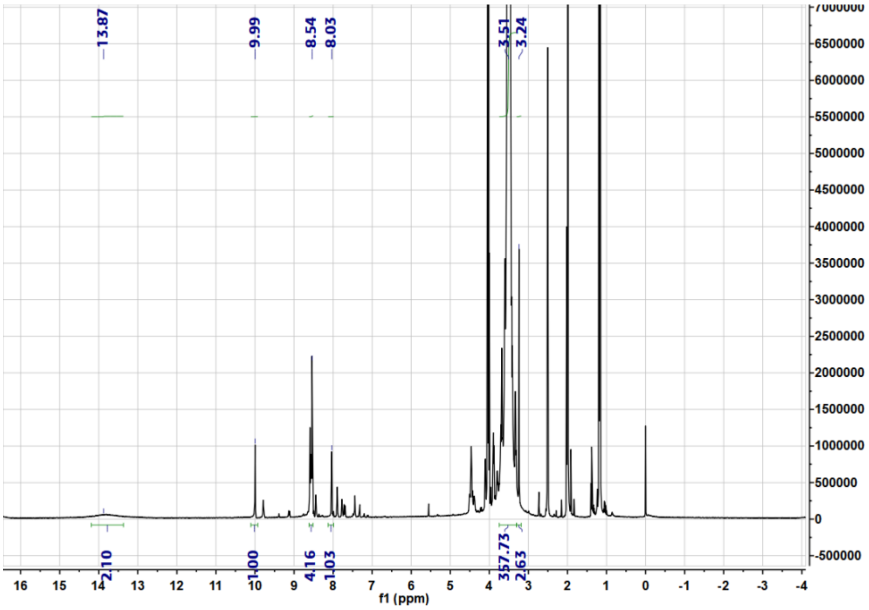

Supplementary Figure 4. ^{1}H NMR spectrum of PEG-Im-BDC in DMSO-d_6 (r.t).

2.3 Synthesis of *p*-*tert*-butylsulfonylcalix[4]arenes (H_4TBSC):

The *p-tert*-butylsulfonylcalix[4]arenes (H_4TBSC) was synthesized according to the literature[3-5].

(1) Synthesis of *p-tert*-Butylthiacalix[4]arene

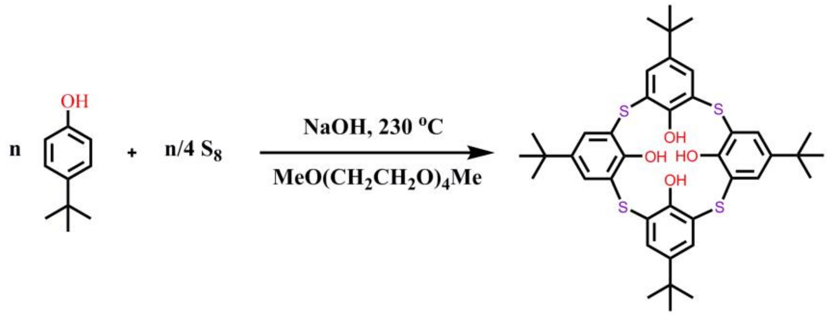

Supplementary Figure 5. Synthesis of *p*-tert-Butylthiacalix[4]arene.

A mixture of *p-tert*-butylphenol (64.5 g, 0.43 mol), elemental sulfur S_8 (27.5 g, 0.86 mol), and NaOH (8.86 g, 0.215 mol) in tetraethylene glycol dimethyl ether (19 mL) was stirred under nitrogen. The stirred mixture was heated gradually to 230 ^oC over a period of 4 h and kept at this temperature for further 3 h with concomitant removal of the evolving hydrogen sulfide with a slow stream of nitrogen. The resulting dark red product was cooled to ambient temperature and diluted with toluene (35 mL) and 4 M aq. sulfuric acid solution (140 mL), followed by addition of diethyl ether (140 mL) with stirring to give a suspension. The precipitate was collected by filtration, recrystallized from chloroform and dried in vacuo (100 ^oC, 4 h) to give an essentially pure sample of *p*-tert-Butylthiacalix[4]arene as pink powder(37.9 g, 49% based on the *p-tert*-butylphenol). The mother liquor of the recrystallization was concentrated in vacuo, and chromatography of the residue on silica gel (hexane/$CHCl_3$ = 4:6) afforded additional *p*-tert-Butylthiacalix[4]arene (3.9 g, 5%), the combined yield of *p*-tert-Butylthiacalix[4]arene amounting to 54% yield (41.8 g,

0.058 mol). ^{1}H NMR (400 MHz, $CDCl_3$, ppm): δ 9.60 (s, 4H), 7.64 (s, 8H), and 1.22 (s, 36H). ^{13}C NMR (400 MHz, $CDCl_3$, ppm): δ = 155.6, 143.7, 136.4, 120.5, 34.2, 31.3. HRMS(ESI) m/z: $[M]^+$ calculated for $C_{40}H_{48}O_4S_4$, 720.2135; found, 720.2136. Elemental analysis (calculated, found for $C_{40}H_{48}O_4S_4$): C (66.63, 66.87), H (6.71, 6.58).

(2) Synthesis of *p-tert*-Butylsulfonylcalix[4]arene

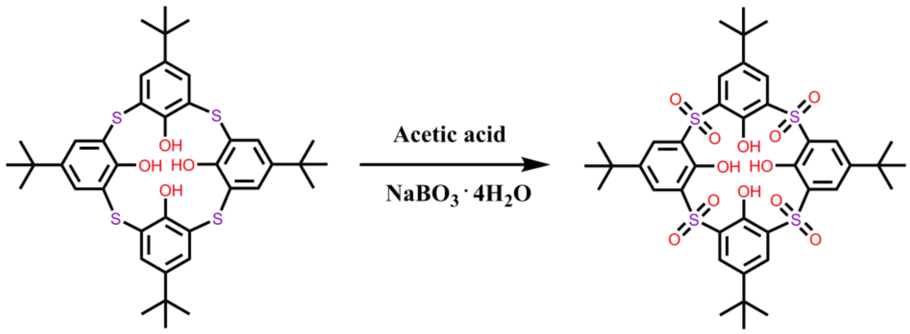

Supplementary Figure 6. Synthesis of *p-tert*-Butylsulfonylcalix[4]arene.

To a solution of *p-tert*-Butylthiacalix[4]arene (1.0 g, 1.38 mmol) in $CHCl_3$ (30 mL) were added acetic acid (50 mL) and $NaBO_3 \cdot 4H_2O$ (2.0 g, 13 mmol). The mixture was stirred at 50 ^oC for 18 h. Upon cooling, 30 mL of H_2O was added. The reaction product was then extracted with chloroform (30 mL × 3). After a drying step with $MgSO_4$, the chloroform solution was evaporated to dryness to give rise to the crude product. The produce was recrystallized from benzene-methanol and dried in vacuo (70 ^oC, 12 h) to give the off-white product of p-tert-Butylsulfonylcalix[4]arene (1.06 g, 1.25 mmol, 90.6% yield). ^{1}H NMR (400 MHz, $CDCl_3$, ppm): δ 7.99 (s, 8H), 1.25 (s, 36H). ^{13}C NMR (400 MHz, $CDCl_3$, ppm): δ = 153.2, 141.7, 132.6, 124.3, 34.8, 31.5. HRMS(ESI) m/z: $[M]^+$ calculated for $C_{40}H_{48}O_{12}S_4$, 848.2029; found, 848.2025. Elemental analysis (calculated, found for $C_{40}H_{48}O_{12}S_4$): C (56.58, 56.13), H (5.71,

5.89).

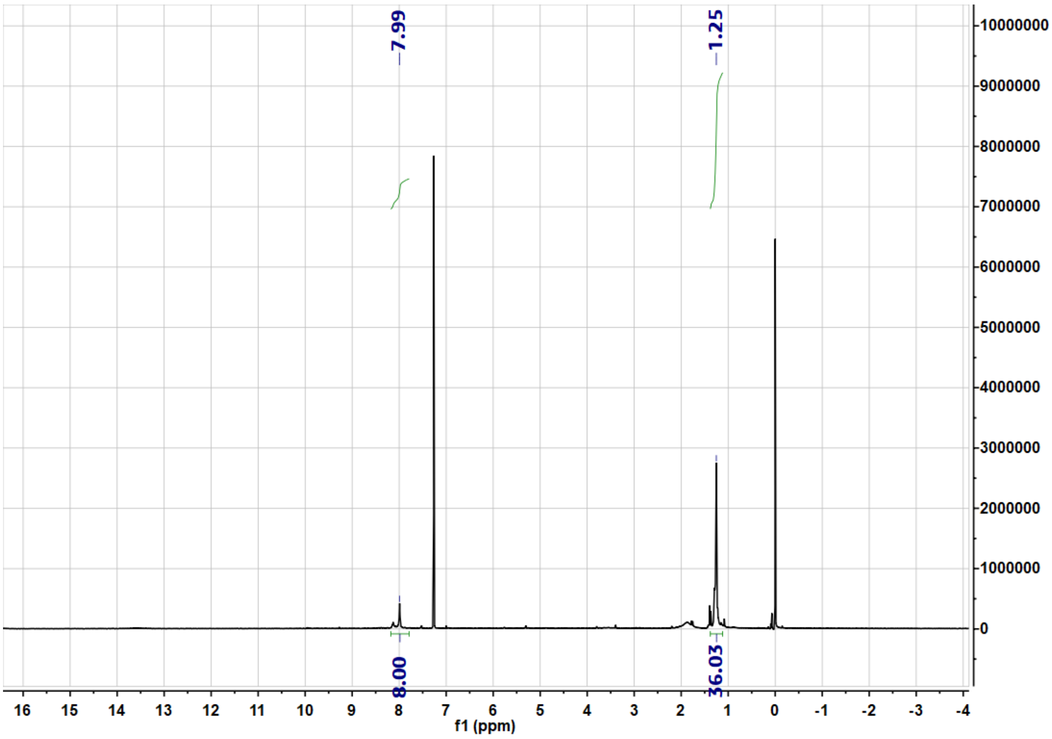

Supplementary Figure 7. ^{1}H NMR spectrum of *p*-*tert*-Butylsulfonylcalix[4]arene in $CDCl_3$ (r.t).

2.4 Synthesis of metal-organic cages:

(1) Zn-Cage: $Zn(NO_3)_2$ $6H_2O$ (74.4 mg, 0.25 mmol), $[(5\text{-Meim-1,3-}H_2BDC)^+(Cl^-)]$ (41.1 mg, 0.11 mmol) and TBSC (42.5 mg, 0.05 mmol) were dissolved in 10 mL of *N*,*N*-dimethylformamide (DMF) and 5 mL of methanol in a scintillation vial (20 mL capacity). The vial was placed in a sand bath, which was transferred to a programmable oven and heated at a rate of 0.5 ^oC /min from 35 to 100 ^oC. The temperature was held at 100 ^oC for 24 h before the oven was cooled at a rate of 0.2 ^oC /min to a final temperature of 35 ^oC. Yellow crystals of Zn-Cage that formed in 5 days were isolated by washing with methanol and dried in the air to give 63.5 mg of the as-synthesized material ^{1}H NMR (400 MHz, DMSO-d_6, ppm): δ 9.37 (s, 1H), 8.46 (s,

1H), 8.23 (m, 3H), 8.13 (s, 1H), 7.94 (m, 4H), 3.90 (s, 3H), 1.22 (s, 18H). ^{13}C NMR (400 MHz, DMSO-d_6, ppm): δ = 175.3, 151.8, 140.9, 136.7, 132.4, 128.9, 124.1, 35.2, 31.6. HRMS(ESI) m/z: $[M]^+$ calculated for Zn-Cage, 1080.1473 1352.1285, 1689.0412, 2788.1227; found, 1080.1472 1352.1286, 1689.0410, 2788.1226. Elemental analysis (calculated, found for Zn-Cage): C (45.31, 44.87), H (3.42, 3.75), N (3.37, 3.56).

(2) Im-PL-Cage: $Zn(NO_3)_2 \cdot 6H_2O$ (74.4 mg, 0.25 mmol), PEG-Im-BDC (473 mg, 0.11 mmol) and TBSC (42.5 mg, 0.05 mmol) were dissolved in 10 mL of *N*,*N*-dimethylformamide (DMF) and 5 mL of methanol in a scintillation vial (20 mL capacity). The vial was placed in a sand bath, which was transferred to a programmable oven and heated at a rate of 0.5 ^{o}C/min from 35 to 100 ^{o}C. The temperature was held at 100 ^{o}C for 24 h before the oven was cooled at a rate of 0.2 ^{o}C /min to a final temperature of 35 ^{o}C. The cage was precipitated out of solution via the addition of excess diethyl ether and centrifuged. The resulting oil was concentrated under reduced pressure yielding a dark yellow, viscous liquid Im-PL-Cage (378 mg). ^{1}H NMR (400 MHz, DMSO-d_6, ppm): δ 9.76 (s, 1H), 8.34 (m, 4H), 7.92 (m, 5H), 3.48 (m, 360H), 3.15 (s, 3H), 1.21 (s, 18H). ^{13}C NMR (400 MHz, DMSO-d_6, ppm): δ = 170.8, 151.6, 140.7, 134.8, 131.5, 125.0, 122.7, 78.6, 72.3, 69.6, 53.8, 35.2, 31.5. HRMS(ESI) m/z: $[M]^+$ calculated for Im-PL-Cage, 1197.1918, 1827.2885, 2503.9657, 3077.5915; found, 1197.1914, 1827.2886, 2503.9659, 3077.5916. Elemental analysis (calculated, found for Im-PL-Cage): C (52.80, 53.23), H (8.07, 8.05), N (0.58, 0.62).

Section 3. Catalyses

Details of the hydrosilylation of CO_2 to formamides：

In a typical procedure, the Im-PL-Cage (300 mg) was placed in an autoclave at 298 K. After being sealed, the autoclave was purged thrice with CO_2 and filled with 20 bar CO_2. Subsequently, the autoclave was rapidly freezed by liquids nitrogening for 3 min. Excess pressure was released to 1 bar, opening the autoclave to exclude the rest CO_2. Afterward, the morpholine (0.87 mL, 10 mmol), phenylsilane (2.47 mL, 20 mmol) and 5ml DMF were added to the autoclave. After being sealed again, the reaction was carried out at 60 ℃ for 10 h. After the reaction completed, small amounts of the mixture was withdrew, diluted with ethanol and filtered with a 0.22 μm membrane filter. The product was determined by gas chromatography. All the control experiments were carried out under the same reaction conditions with different carbon dioxide adsorbents.

Section 4. Additional Figures and Tables

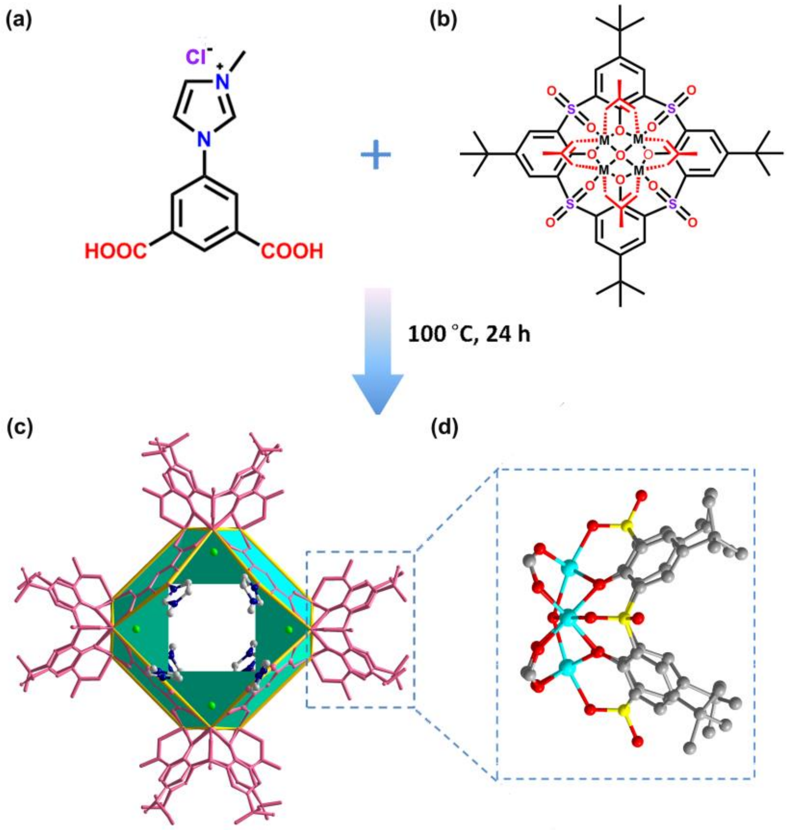

Supplementary Figure 8. The scheme for the self-assembly of Zn-Cage from $[(5\text{-Meim-1,3-}H_2BDC)^{+}(Cl^{-})]$, H_4TBSC and M_4-TBSC (M=Zn). **a** Structures of the $[(5\text{-Meim-1,3-}H_2BDC)^{+}(Cl^{-})]$ linker and **b** calix[4]arene-based metal node and **c** The single-crystal X-ray structure of the Zn-Cage, the yellow lines and blue polyhedron are used to highlight the shape of cage. **d** The tetranuclear $[Zn_4(\mu_4\text{-OH})(TBSC)]$ unit.

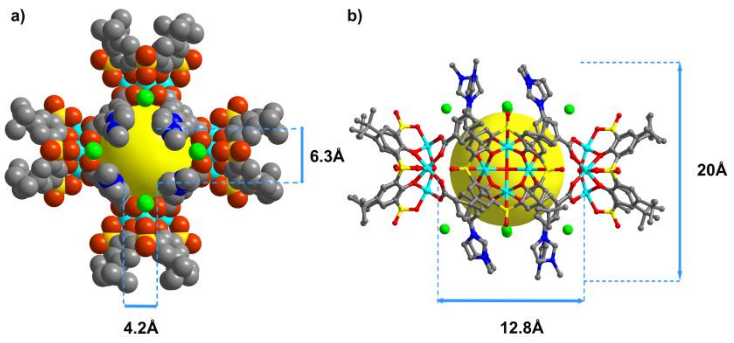

Supplementary Figure 9. Crystal structure of Zn-Cage. **a** space filling views of the Zn-Cage. **b** ball-and-stick views of the Zn-Cage. (Color code: Zn, blue; S, yellow; O, red; C, gray; N, blue; Cl, green), H atoms are omitted for clarity.

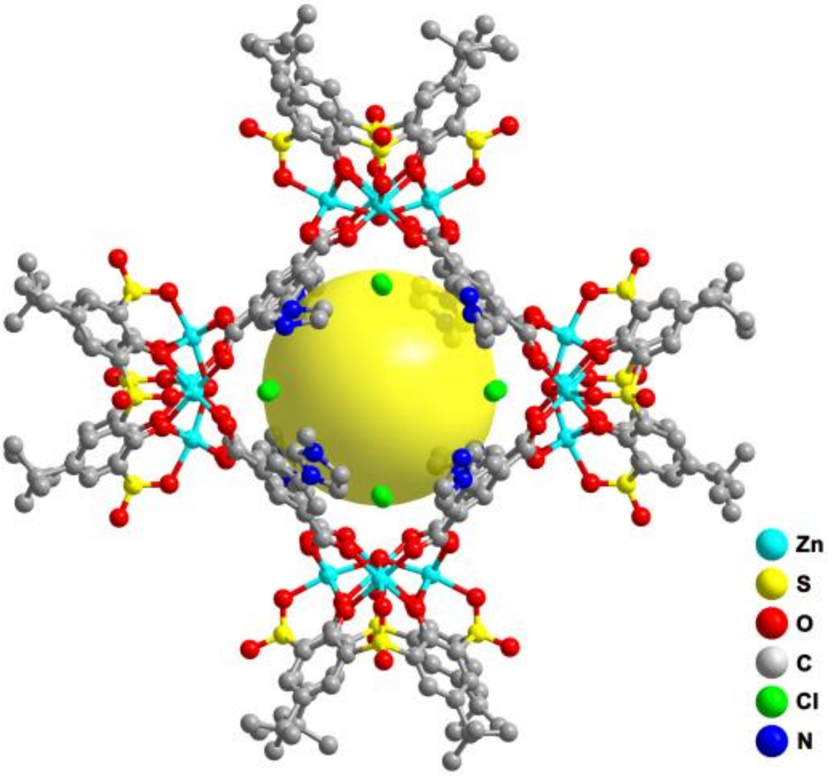

Supplementary Figure 10. Chemical structure of a cage in Zn-Cage. H atoms are omitted for clarity.

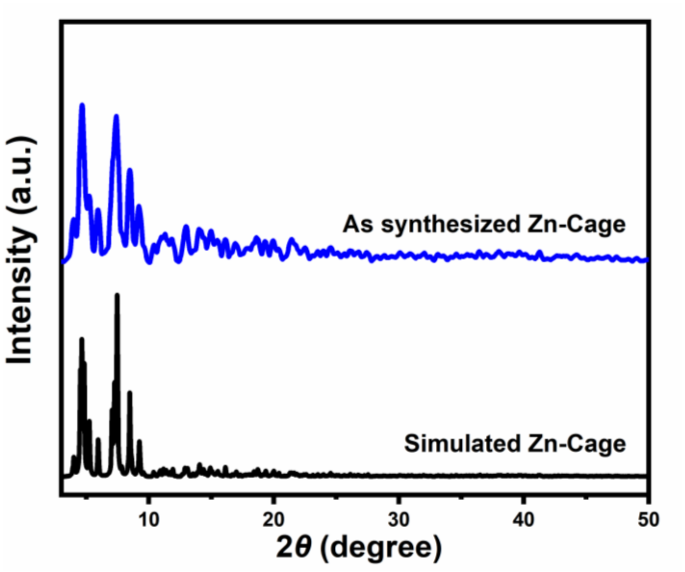

Supplementary Figure 11. PXRD patterns for Zn-Cage.

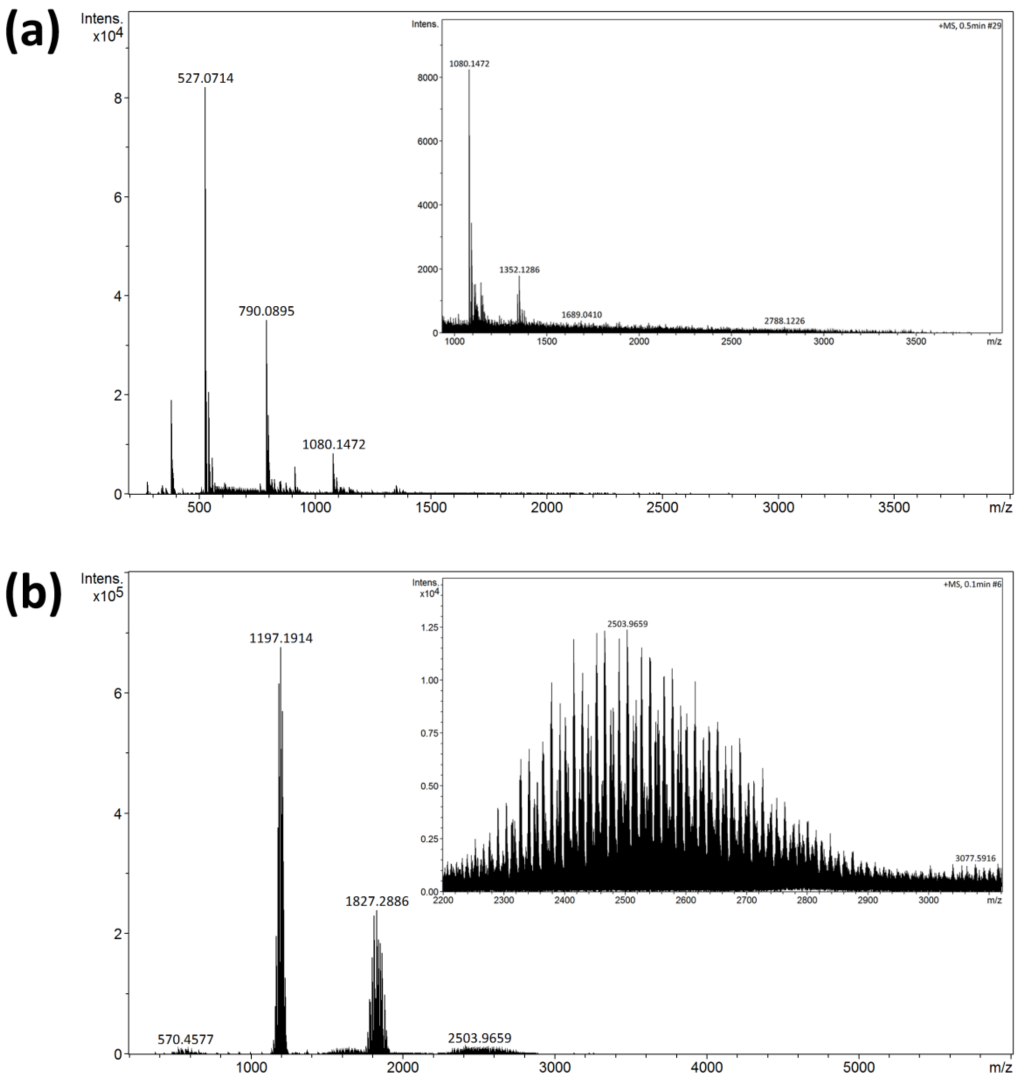

Supplementary Figure 12. MS spectra of **a** Zn-Cage and **b** Im-PL-Cage.

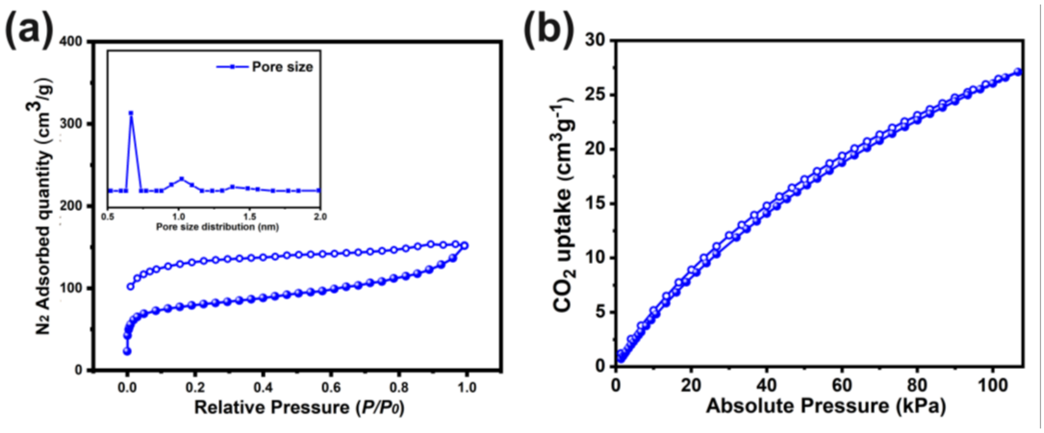

Supplementary Figure 13. **a** N_2 sorption isotherms of Zn-Cage at 77 K (inset pore-size distribution profile). **b** CO_2 sorption isotherm of Zn-Cage at measured at 298 K.

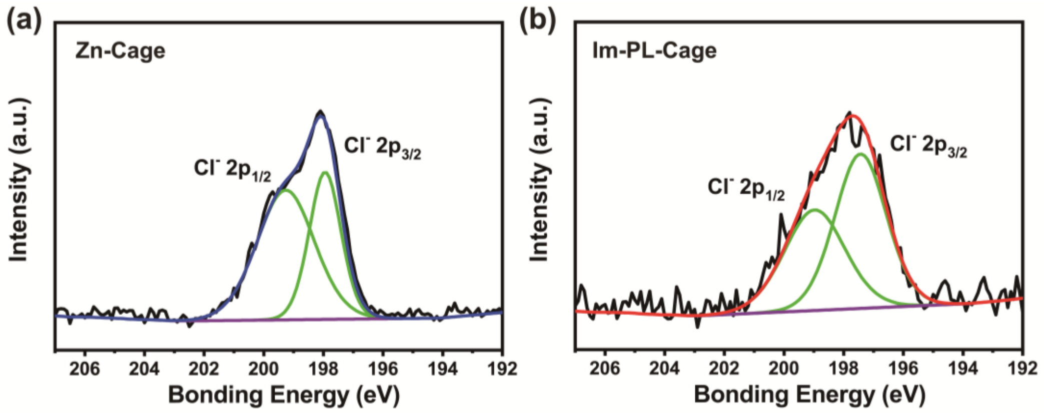

Supplementary Figure 14. XPS survey, Cl 2*p* spectra of **a** Zn-Cage, **b** Im-PL-Cage.

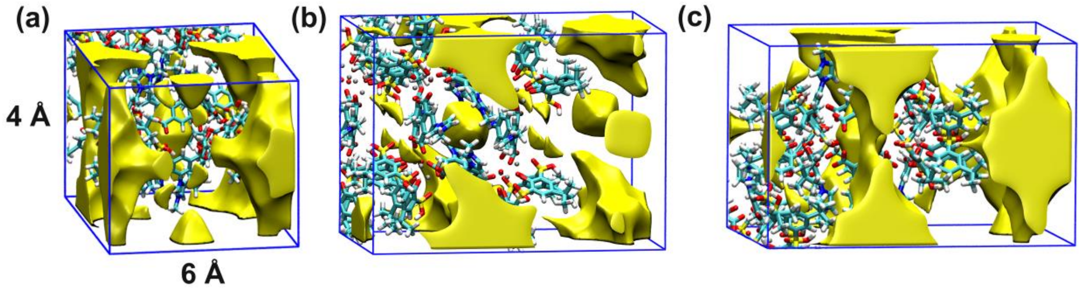

Supplementary Figure 15. The snapshots of simulation boxes for Im-PL-Cage and

pore space in Im-PL-Cage. The views of the **a** X, **b** Y and **c** Z axes, respectively.

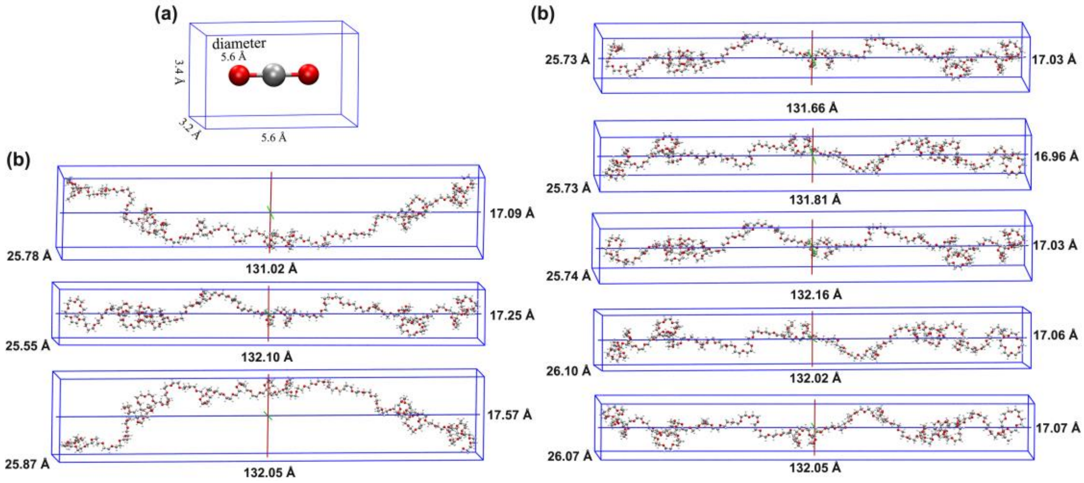

Supplementary Figure 16. The sizes of (a) CO_2 and (b) PEG chains calculated by density functional theory (DFT).

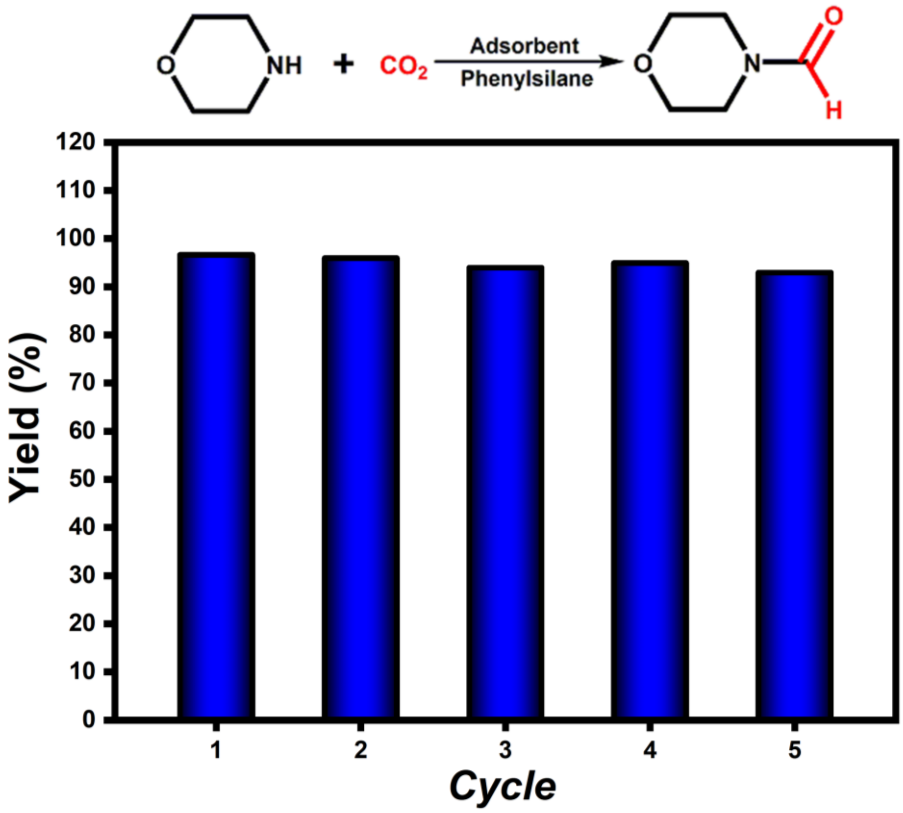

Supplementary Figure 17. The recyclability test of Im-PL-Cage.

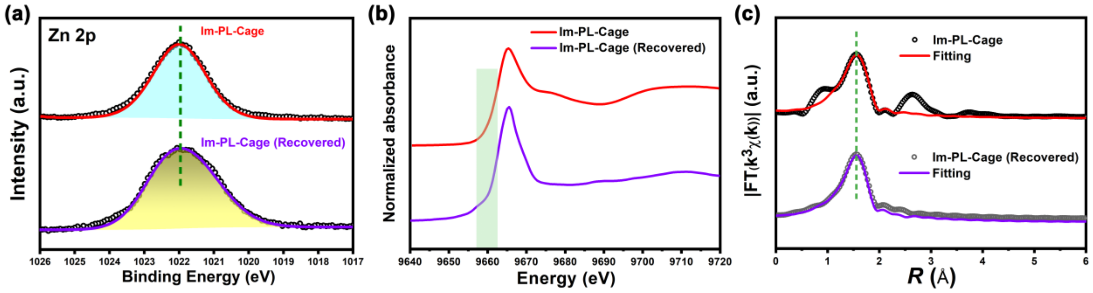

Supplementary Figure 18. a The XPS Zn 2*p* spectra of Im-PL-Cage and Im-PL-Cage (Recovered). **b** The normalized Zn K-edge XANES spectra of Im-PL-Cage and Im-PL-Cage (Recovered). **c** Fourier transform EXAFS fitting curves of Zn K-edge for Im-PL-Cage and Im-PL-Cage (Recovered).

Supplementary Table 1. Crystal data and structure refinement for Zn-Cage.

Identification code	**Zn-Cage**
CCDC number	2259420
Empirical formula	$C_{251}H_{226}N_{16}O_{84}Cl_8S_{16}Zn_{16}$
Formula weight	6653.45
Temperature/K	273
Crystal system	Triclinic
Space group	$P\bar{1}$
Unit cell dimensions	a = 23.4907(16) Å<br>b = 24.8315(16) Å<br>c = 25.4489(15) Å<br>α = 72.449(3) °<br>β = 63.420(3) °<br>γ = 63.343(3) °
Volume (Å^3)	11769.8(14)
Z	2
Density (calculated) (g·cm^3)	1.039
Absorption coefficient (mm^{-1})	1.036
F(000)	6837
Crystal size (mm^3)	0.2×0.2×0.2
Reflections collected	336592
Independent reflections	24865 [R_{int} = 0.1142, R_{sigma} = 0.0462]
Goodness-of-fit on F^2	1.061
Final R indices [I>2σ(I)]	R_1 = 0.0932, wR_2 = 0.2412
Final R indices (all data)	R_1 = 0.1333, wR_2 = 0.2803

Supplementary Table 2. ICP and EA of Zn-Cage and Im-PL-Cage.

		Zn	C	H	N	S
Zn-Cage (Calculated)	ICP	15.73 %				
	EA		45.31 %	3.42 %	3.37 %	7.71%
Zn-Cage (Found)	ICP	14.36 %				
	EA		44.87 %	3.75 %	3.56 %	7.81 %
Im-PL-Cage (Calculated)	ICP	2.72 %				
	EA		52.80 %	8.07 %	0.58 %	1.33%
Im-PL-Cage (Found)	ICP	2.67 %				
	EA		53.23 %	8.05 %	0.62 %	1.35%

Supplementary Table 3. Fitting results from EXAFS analysis of Im-PL-Cage.

Sample	Path	CN	R(Å)	σ^2(10^{-3}Å^2)	R factor
Im-PL-Cage	Zn-O	6	2.03	9.9±0.6	0.005
Im-PL-Cage (Recovered)	Zn-O	6	2.03	4.7±1.2	0.005

CN, coordination number; R, distance between absorber and backscatter atoms; σ^2, Debye-Waller factor (a measure of thermal and static disorder in absorber-scatterer distances); R factor is used to value the goodness of the fitting.

Supplementary Table 4. The hydrosilylation of CO_2 to formamides.

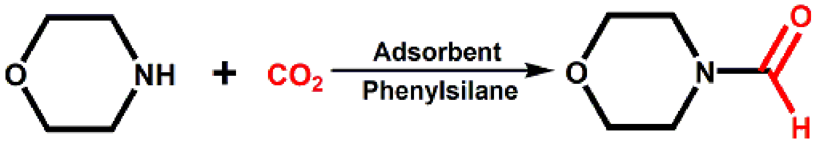

Entry	Adsorbent	Yield (%)	N-formylmorpholine (mmol)	Adsorption of CO_2 (mmol)
1	Blank[a]	16.3	1.63	-
2	PEG-Im-H_2BDC (1.64 g)	21.5	2.15	0.52
3	Im-PL-Cage (2 g)	96.7	9.37	7.74
4	Zn-Cage (0.43 g)	24.5	2.45	0.82
5	Zn-Cage (0.43 g) + PEG-Im-H_2BDC (1.57 g)	31.9	3.19	1.56

Reaction conditions: CO_2 was adsorbed and stored in Im-PL-Cage until the maximum uptake in the autoclave with 20 bar CO_2. Then, the valve of the autoclave was opened until the pressure was balanced with atmospheric pressure. The morpholine (0.87 mL, 10 mmol), phenylsilane (2.47 mL, 20 mmol) and 5ml DMF were added into the autoclave to react with CO_2 that stored and released from Im-PL-Cage at 60 °C.

[a] The hydrosilylation of CO_2 to formamides can occur spontaneously and slowly in DMF[6].

Supplementary Table 5. The hydrosilylation of CO_2 to formamides.

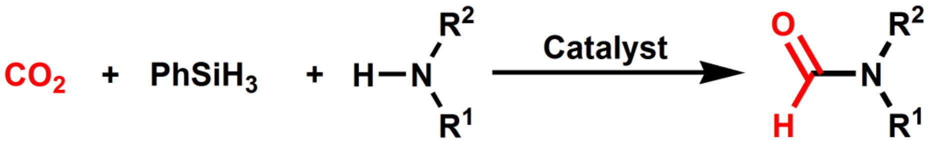

Entry	Substrate	Conversion[b]	Selectivity[b]
1	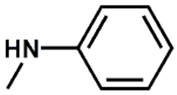	97%	99%
2	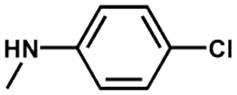	88%	95%
3	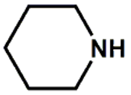	96%	99%
4	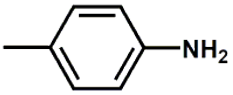	91%	97%
5	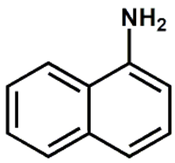	85%	93%

Reaction conditions: CO_2 was adsorbed and stored in 2 g Im-PL-Cage until the maximum uptake in the autoclave with 20 bar CO_2. Then, the valve of the autoclave was opened until the pressure was balanced with atmospheric pressure. The amines (10 mmol), phenylsilane (20 mmol) and 5ml DMF were added into the autoclave to react with CO_2 that stored and released from Im-PL-Cage at 60 °C.

Section 5. References

1. Zhu, S. L. et al. A porous metal-organic framework containing multiple active Cu^{2+} sites for highly efficient cross dehydrogenative coupling reaction. *Dalton Trans*. **44**, 2038-2041 (2015).

2. Cheng, F. J. et al. Fine-tuning optimal porous coordination polymers using functional alkyl groups for CH_4 purification. *J. Mater. Chem. A*. **5**, 17874-17880 (2017).

3. Lki, N. et al. Synthesis of p-tert-Butylthiacalix[4]arene and its inclusion property. *Tetrahedron.* **56**, 1437-1443 (2000).

4. Lki, N. et al. Selective oxidation of thiacalix[4]arenes to the sulfinyl- and sulfonylcalix[4]arenes and their coordination ability to metal ions. *Tetrahedron Letters.* **39**, 7559-7562 (1998).

5. Morohashi, N., Iki, N., Sugawara, A. & Miyano, S. Selective oxidation of thiacalix[4]arenes to the sulfinyl and sulfonyl counterparts and their complexation abilities toward metal ions as studied by solvent extraction. *Tetrahedron.* **57**, 5557-5563 (2001).

6. Lv, H. et al. Solvent-promoted catalyst-free n-formylation of amines using carbon dioxide under ambient conditions. *Chem. Commun.* **52**, 6545-6548 (2016).
