# Supplementary material for: A porous metal-organic cage liquid for sustainable CO2 conversion reactions
Source: Nat Commun. 2023 Jun 7;14:3317. doi: 10.1038/s41467-023-39089-x (PMC10247695; doi:10.1038/s41467-023-39089-x)
Supplement: Supplementary file 3 — Description of Additional Supplementary Files [file 41467_2023_39089_MOESM3_ESM.pdf]

Description of Additional Supplementary Files

File Name: Supplementary Movie 1
Description: Fluidity of Im-PL-Cage.
